# Supplementary material for: Controversial Aspects of Diagnostics and Therapy of Arthritis of the Temporomandibular Joint in Rheumatoid and Juvenile Idiopathic Arthritis—An Analysis of Evidence- and Consensus-Based Recommendations Based on an Interdisciplinary Guideline Project
Source: J Clin Med. 2022 Mar 22;11(7):1761. doi: 10.3390/jcm11071761 (PMC8999183; doi:10.3390/jcm11071761)
Supplement: Supplementary file 1 [file jcm-11-01761-s001.zip › Supplement Table S1&2.pdf]

Table S1: Members of the working group „oral and maxillofacial surgeons" (OMFS) of the German Association for Oral and Maxillofacial Surgery (Deutsche Gesellschaft für Mund-, Kiefer- und Gesichtschirurgie - DGMKG)

Depratment/Clinic/Medical Practice	Members
Klinik und Poliklinik für Mund-, Kiefer- und Gesichtschirurgie, Universitätsklinikum Marburg, Baldingerstraße 35043, Marburg	Prof. Dr. med. Dr. med. dent. Prof. h.c. (BNMU, Kyiv) Andreas Neff
Klinik für Mund-, Kiefer- und Gesichtschirurgie<br>Universitätsklinikum Münster<br>Albert-Schweitzer-Campus 1<br>48149 Münster	Prof. Dr. med. Dr. med. dent. Johannes Kleinheinz
Universitätsklinik für Mund-, Kiefer- und Gesichtschirurgie<br>Universitätskliniken/Tirol Kliniken<br>Anichstraße 35<br>A-6020 Innsbruck	Prof. Dr. med. Dr. med. dent. Andreas Kolk
Medizin & Ästhetik, Praxisklinik für Mund-, Kiefer- und Gesichtschirurgie<br>Lenbachplatz 2a<br>Alte Börse<br>80333 München	Prof. Dr. med. Dr. med. dent. Christoph Pautke
MVZ R(h)einZahn<br>Welschnonnenstraße 1-5<br>53111 Bonn	Prof. Dr. med. Dr. med. dent. Rudolf Reich
Praxis für Gesichtschirurgie und Kiefergelenkchirurgie<br>Brockdorfstr. 90<br>22149 Hamburg	Dr. med. Dr. med. dent. Marcus Teschke

Table S2: Participating medical Expert Associations and Patient Associations including the elected Representatives

Association	Elected Representative
Deutsche Gesellschaft für Mund-, Kiefer-, Gesichtschirurgie (DGMKG)	Prof. Dr. med. Dr. med. dent. Prof. h.c. (BNMU, Kyiv) Andreas Neff
Deutschen Gesellschaft für Neuroradiologie (DGNR)	Priv.-Doz. Dr. med. Tim Hilgenfeld
Deutsche Gesellschaft für Orthopädie und Orthopädische Chirurgie (DGOOC)	Dr. med. Martin Arbogast
Deutsche Gesellschaft für Zahn-, Mund- und Kieferheilkunde (DGZMK)	
- Arbeitsgemeinschaft für Oral- und Kieferchirurgie (AGOKi)	Dr. med. Dr. med. dent. Andreas Schön
- Deutsche Gesellschaft für Funktionsdiagnostik und -therapie (DGFDT)	Priv.-Doz. Dr. med. dent. Oliver Ahlers
- Deutsche Gesellschaft für Kieferorthopädie (DGKFO)	Prof. Dr. med. dent. Bernd Koos
- Deutsche Gesellschaft für Kinderzahnheilkunde (DGKiZ)	Prof. Dr. med. dent. Christian Hirsch
- Deutsche Gesellschaft für Prothetische Zahnmedizin und Biomaterialien (DGPro)	Prof. Dr. med. dent. Peter Ottl
Deutsche Röntgengesellschaft (DRG)	Priv.-Doz. Dr. med. Wolfgang Wüst
Deutscher Verband für Physiotherapie (ZVK)	Ima Feurer
Gesellschaft für Kinder- und Jugendrheumatologie (GKJR)	Dr. med. Boris Hügle
Gesellschaft für Pädiatrische Radiologie (GPR)	Priv.-Doz. Dr. med. Thekla von Kalle
**Patientenverband**	**Mandatsträger**
Deutsche Rheuma-Liga: Bundesverband	Mario Habermann-Krebs
