# Supplementary material for: Controversial Aspects of Diagnostics and Therapy of Arthritis of the Temporomandibular Joint in Rheumatoid and Juvenile Idiopathic Arthritis—An Analysis of Evidence- and Consensus-Based Recommendations Based on an Interdisciplinary Guideline Project
Source: J Clin Med. 2022 Mar 22;11(7):1761. doi: 10.3390/jcm11071761 (PMC8999183; doi:10.3390/jcm11071761)
Supplement: Supplementary file 1 [file jcm-11-01761-s001.zip › Supplement Table S3 - Basistabelle.pdf]

Table S3: Voting results of the Delphi process

(Abbreviations: BSSO: bilateral sagittal split osteotomy, CBCT: cone beam computed tomography, CMD: craniomandibular dysfunction, CPAP: continuous positive airway pressure, CT: computed tomography, DGMKG: Deutsche Gesellschaft für Mund-, Kiefer- und Gesichtschirurgie - German Association for Oral and Maxillofacial Surgery, IACI: intraarticular corticosteroid injection, JIA: juvenile idiopathic arthritis, MRI: magnetic resonance imaging, NSAID: nonsteroidal anti-inflammatory drug, OMERACT: initially "Outcome Measures in Rheumatoid Arthritis Clinical Trials" changed to „Outcome Measures in Rheumatology", OMFS/OMS: oral and maxillofacial surgeon, OPG: orthopantomography, PAS: posterior airway space, TMJ: temporomandibular joint)

	Recommendation/Statement	Applicable for	LoE	GoR	1/1	2/1	3/1	1/2	2/2	3/2
1.	The TMJaw working group's examination protocol provides a relatively fast and easily executed option for initial assessment of the craniomandibular status in JIA patients.	JIA/RA[1]	IIIb	Statement					**Strong consensus (100%)**<br>**"yes" 12/12, abstentions: 1**	
2.	Clinical evaluation for initial assessment of the craniomandibular status in JIA patients **should** be performed according to the TMJaw working group's examination protocol.	JIA	IIIb	B				**Strong consensus (100%)**<br>**„should" 11/11[2], abstentions: 2**		
3.	For diagnosis of TMJ arthritis, a clinical examination alone is insufficient and **shall** be complemented by medical imaging.	JIA/RA	IIb	A	**Strong consensus (100%)**<br>**"shall" 6/6**			**Strong consensus (100%)**<br>**"shall" 13/13**		
4.	Nevertheless, a clinical examination is basis of the clinical management of chronic rheumatic arthritis of the temporomandibular joint – this is true especially regarding the question if medical imaging is indicated and in the context of clinical monitoring and follow-up.	JIA/RA	IIIb	Statement					**Strong consensus (100%)**<br>**"yes" 13/13**	
5.	In JIA patients in childhood and adolescence, the temporomandibular joint **shall** be monitored clinically in regular intervals.	JIA	IV	Expert consensus	**Strong consensus (100%)**<br>**"shall" 6/6**			**Strong consensus (100%)**<br>**"shall" 13/13**		
6.	In JIA patients with TMJ involvement, interdisciplinary follow-ups of the craniomandibular status **shall** be continued beyond childhood and adolescence (≥ 18 years of age).	JIA	IIb	A					**Strong consensus (100%)**<br>**"shall" 12/12, abstentions: 1**	
7.	As it enables detection of acute inflammatory processes in soft tissue, contrast-enhanced MRI constitutes the current state-of-the art method for imaging of active arthritis of the temporomandibular joint[3].	JIA/RA	IIb	Statement	**Strong consensus (100%)**<br>**"yes" 6/6**			**Strong consensus (100%)**<br>**"yes" 13/13**		
8.	In the context of arthritis of the temporomandibular joint in JIA, contrast-enhanced MRI is the medical imaging method of choice both for early diagnosis and monitoring of therapy progress[4].	JIA	IIb	Statement	**Strong consensus (100%)**<br>**"yes" 6/6**			**Strong consensus (100%)**<br>**"yes" 13/13**		

[1] Representative of TMJ arthritis in the context of chronic rheumatic disorders in adulthood
[2] Two voting members out of the 11/11 who voted in favour of "should" also voted in favour of "shall"
[3] For the detection of osseous structures of the TMJ, MRI is considered inferior to CT and CBCT (Bag et al. 2014, V/k+). For further details, please refer to AWMF S2k Guideline No. 083/005 "Dental Volume Tomography".
[4] The AWMF S2k Guideline No. 083/005 "Dental Volume Tomography" contains a similar assessment

	Recommendation/Statement	Applicable for	LoE	GoR	1/1	2/1	3/1	1/2	2/2	3/2
9.	Due to the largely subjective/ qualitative evaluation of MRI files and the lack of standardized uniform criteria for diagnosis of TMJ arthritis by means of MRI, such standardization of criteria is urgently needed for quantitative, objectifiable assessment. The OMERACT group and Resnick and colleagues have presented promising approaches for this purpose. In order to be able to develop general recommendations, additional validating studies are required.	JIA/RA	IIIb	Statement					**Strong consensus (100%)**<br><br>**"yes" 13/13**	
10.	It must be emphasized that there remains great need for a practicable screening tool suitable for daily clinical routine.	JIA/RA	IV	Statement		**Strong consensus (100%)**<br><br>**"yes" 6/6**		**Strong consensus (100%)**<br><br>**"yes" 13/13**		
11.	In cases of suspected chronic-rheumatic TMJ arthritis, OPG provides a cost-efficient, low-risk and widely available method for initial medical imaging for detection of advanced bony involvement of the temporomandibular joint[5].	JIA/RA	IIb	Statement	**Strong consensus (100%)**<br><br>**"yes" 6/6**			**Consensus (77%)**<br><br>**"yes" 10/13, "no" 3/13**<br><br>Change in wording (based on comments of two voting members and after an initial simple consensus)	**Strong consensus (100%)**<br><br>**"yes" 13/13**	
12.	OPG, however, is not suitable for routine screenings for TMJ involvement in patients with a chronic rheumatic underlying disorder, due to the limited visibility of lower-degree bony lesions and its inability to detect active arthritis.	JIA/RA	IIb	Statement					**Strong consensus (100%)**<br><br>**"yes" 13/13**	
13.	Lateral X-ray imaging including cephalometric analysis constitutes an option for orthodontic assessment of disturbance of mandibular growth and monitoring of dentofacial growth in JIA patients.	JIA/RA	IV	Statement	**Strong consensus (100%)**<br><br>**"yes" 6/6**			**Strong consensus (100%)**<br><br>**"yes" 13/13**		
14.	Should MRI imaging offer insufficient information on the involvement of bony structures in chronic rheumatic TMJ arthritis, CT offers a possible alternative option.	JIA/RA	IIb	Statement	**Strong consensus (100%)**<br><br>**"yes" 6/6**			**Strong consensus (100%)**<br><br>**"yes" 13/13**		
15.	Due to insufficient imaging quality of soft tissue structures and of the early stages of arthritis of the temporomandibular joint, and the radiation load (as compared to MRI), CT and CBCT are not suitable as routine methods in cases of chronic rheumatic arthritis of the temporomandibular joint.	JIA/RA	IIIb	Statement	**Strong consensus (100%)**<br><br>**"yes" 6/6**			**Strong consensus (100%)**<br><br>**"yes" 13/13**		
16.	CBCT **may** be used for evaluation of bony structures of the temporomandibular joint in chronic rheumatic TMJ arthritis as potential dosage-efficient alternative to CT. Radiation exposure will largely depend on choice of device and examination protocol.	JIA/RA	IV	0	**Strong consensus (100%)**<br><br>**"may" 6/6**			**Consensus (85%)**<br><br>**"may" 11/13, dissenting votes 2/13**<br><br>Change in wording (based on comments by three voting members)	**Strong consensus (100%)**<br><br>**"may" 13[6]/13**	
17.	Three-dimensional cephalometric analysis by CBCT is an option for true-to-scale assessment of bony cranial morphology and thus for assessment of possible growth disturbances in JIA with TMJ involvement.	JIA/RA	IIIb	Statement	**Strong consensus (100%)**<br><br>**"yes" 6/6**			**Strong consensus (100%)**<br><br>**"yes" 12/12, abstentions: 1**		

[5] Regarding physiological alterations in size and shape of the condyle, especially in childhood (≤ 14 years of age), it is important to take into account the age-specific standard findings for radiological evaluation Karlo et al. 2010, IV.

[6] Two voting members out of the 13/13 who voted in favour of "may" also voted in favour of "should"

	Recommendation/Statement	Applicable for	LoE	GoR	1/1	2/1	3/1	1/2	2/2	3/2
18.	Sonography is currently not considered a suitable means for diagnosis and monitoring of progress of TMJ arthritis with underlying chronic rheumatic disorder due to lack of standardization and limited availability of studies.	JIA/RA	IIIb	Statement	**Strong consensus (100%)**<br><br>**"yes" 6/6**			**Consensus (91%)**<br><br>**"yes" 10/11, "no" 1/11, abstentions: 2**<br><br>Change in wording (based on comments by three voting members)	**Strong consensus (100%)**<br><br>**"yes" 11/11, abstentions: 2**	
19.	Due to quality of imaging and the considerably less invasive nature of contrast-enhanced MRI, arthrography and video fluoroscopy as a rule are currently no longer routinely used for further clinical diagnostics of chronic rheumatic TMJ arthritis.	JIA/RA	V	Statement	**Strong consensus (100%)**<br><br>**"yes" 6/6**			**Strong consensus (100%)**<br><br>**"yes" 13/13**		
20.	Conventional (2D) photography constitutes an easily accessible, radiation-free option for assessment and monitoring of progress of facial morphology based on soft tissue structures, however, can not replace a cephalometric analysis with radiological methods for assessment of cranial bone morphology and growth.	JIA/RA	IV	Statement				**Strong consensus (100%)**<br><br>**"yes" 12/12, abstentions: 1**		
21.	When using 2D photography for assessment of facial morphology, a standardized methodology (e.g. Fotostat according to Schwarz) for en face, lateral, and oblique-lateral images **should** be applied.	JIA/RA	V	B					**Strong consensus (100%)**<br><br>**"should" 12/12[7], abstentions: 1**	
22.	3D photography offers a promising alternative approach to 2D photography, and possibly also to cephalometric analysis using radiological methods. In order to be able to establish a general recommendation, further validating studies are required.	JIA/RA	IIIb	Statement				**Strong consensus (100%)**<br><br>**"yes" 12/12, abstentions: 1**		
23.	The great sensitivity of bone scintigraphy allows for early detection of bone remodeling processes – however, at the price of specificity. For diagnosis and monitoring of progress of chronic rheumatic arthritis of the temporomandibular joint, it is indeed a third-choice diagnostic device. Its use should be avoided in children and adolescents due to the radiation exposure involved.	JIA/RA	V	Statement	**No consensus**<br><br>**"yes" 1/6, "no" 1/6, abstentions: 4**<br><br>Change in wording (based on comment by one voting member)	**Strong consensus (100%)**<br><br>**"yes" 6/6**		**Strong consensus (100%)**<br><br>**"yes" 13/13**		
24.	The Krenn synovitis score, well established in orthopedics[8], enables the assessment of the severity of synovitis in the context of surgical interventions, and therefore makes a valuable contribution for the purposes of differential diagnostics and therapy design. The suitability of the method for the temporomandibular joint needs yet to be evaluated.	JIA/RA	IIIb	Statement					**Strong consensus (100%)**<br><br>**"yes" 9/9, abstentions: 4**	
25.	Synovialis analysis by means of the Krenn Score[8] **may**, in individual cases, be considered for the purpose of further assessment and differential diagnosis, independent of an intervention otherwise indicated.	JIA/RA	IIIb	0					**Consensus (88%)**<br><br>**"may" 8/9, Dissenting vote: 1/9, abstentions: 4**	**Strong consensus (100%)**<br><br>**"may" 9/9[9], abstentions: 4**

[7] Two voting members out of the 12/12 who voted in favour of "should" votes also voted in favour of "shall"

[8] For analysis of synovial biopsies from smaller-size joints (e.g. TMJ) by means of the Krenn score, the minimum recommended sample diameter is usually 2.5 mm and the minimum recommended number of samples is four. (Najm et al. 2018, IV, IV). Synovial biopsy as a method for assessment of the TMJ is therefore subject to methodical reservations due to the generally small quantity of biopsy material available from arthroscopy.

[9] Two voting members out of the 9/9 who voted in favour of "may" also voted in favour of "should"

	Recommendation/Statement	Applicable for	LoE	GoR	1/1	2/1	3/1	1/2	2/2	3/2
									Change in wording (based on comment by one voting member)	
26.	Synovialis biopsy, if performed independently from an otherwise indicated intervention, **shall** be limited to a very narrow range of indications to avoid not strictly necessary interventions – especially in patients ≤ 17 years of age.	JIA/RA	V	Expert consensus					**Strong consensus (100%)**<br><br>**"shall" 10/10, abstentions: 3**	
27.	Biopsy of components of the masticatory muscles as additional examination method is not considered a useful approach in the context mentioned above.	JIA/RA	V	Statement	**No consensus**<br><br>**"yes" 1/6, "no" 2/6, abstentions: 3/6**			**No consensus**<br><br>**"yes" 4/4, abstentions: 9**		
28.	If clinical indicators point to structural damage in the absence of pain ("silent arthritis of the temporomandibular joint") and in case of borderline MRI-diagnostic findings[10], sampling and examination of synovial fluid from the temporomandibular joint **may** be considered in individual cases in patients >17 years of age.	RA	IIIb	0	**No consensus**<br><br>**Soll 2/3, "may" 1/3, abstentions: 3**<br><br>Change in wording (based on comment by one voting member)	**Strong consensus (100%)**<br><br>**"may" 6/6**		**Strong consensus (100%)**<br><br>**"may" 12/12, abstentions: 1**		
29.	In adult CMD patients (> 17 years of age) with arthrogenous leading symptoms and an anamnestically known rheumatic underlying disorder[11], who are insufficiently responsive to splint and conservative therapy, and borderline findings[12] in MRI diagnostics, sampling and examination of synovial fluid from the temporomandibular joint **may** be considered in individual cases.	RA	IIIb	0		**Strong consensus (100%)**<br><br>**"may" 6/6**		**Strong consensus (100%)**<br><br>**"may" 12/12, abstentions: 1**		
30.	Figure 1: Diagnostic algorithm when "silent arthritis" of TMJ is suspected in JIA and RA patients based on (Stoll et al. 2018, V/k++) and (Alstergren et al. 2018, IIIb/+/k+)	JIA/RA	V	Diagram		**Strong consensus (100%)**<br><br>**"yes" 6/6**		**Strong consensus (100%)**<br><br>**"yes" 13/13**		
31.	Figure 2: Clinical management when TMJ involvement is suspected and RA/JIA is suspected or anamnestically known, based on (Stoll et al. 2018, V/k++) and (Alstergren et al. 2018, IIIb/+/k+)	JIA/RA	V	Diagram		**Strong consensus (100%)**<br><br>**"yes" 6/6**		**Strong consensus (100%)**<br><br>**"yes" 13/13**		
32.	Electromyography provides a possible additional diagnostic tool.	JIA/RA	V	Statement	**No consensus**<br><br>**"no" 2/6, abstentions: 4/6**			**No consensus**<br><br>**"no" 5/5, abstentions: 8**		
33.	Instrumental recording of the movements of the mandible provides a possible additional diagnostic tool.	JIA/RA	IIb	Statement	**No consensus**<br><br>**"no" 1/6, abstentions: 5/6**			**No consensus**<br><br>**"no" 5/7, "yes" 2/7, abstentions: 6**		
34.	An interdisciplinary therapy approach under the lead of a rheumatologist/pediatric rheumatologist, and the involvement of additional medical specialties, as required, such as radiology, dentistry, especially functional diagnostics, orthodontics, OMFS surgery and physiotherapy is fundamental principle of the therapy of chronic rheumatic TMJ inflammation.	JIA/RA	IIIb	Statement	**Strong consensus (100%)**<br><br>**"yes" 6/6**			**Strong consensus (100%)**<br><br>**"yes" 13/13**		

[10] Borderline MRI findings within the meaning of a lower-degree synovial contrast enhancement or a less pronounced joint effusion ("minimally active inflammation"), as it is also found in healthy individuals Stoll et al. 2018, IIIb/-/k+; Angenete et al. 2018, IV/k+.

[11] Chronic rheumatic disorders which may affect the TMJ– particularly rheumatoid arthritis, also psoriatic arthritis, ankylosing spondylitis and more.

[12] Borderline MRI findings within the meaning of a lower-degree synovial contrast enhancement or a less pronounced joint effusion ("minimally active inflammation"), as it is also found in healthy individuals. (Stoll et al. 2018, IIIb/-/k+; Angenete et al. 2018, IV/k+).

	Recommendation/Statement	Applicable for	LoE	GoR	1/1	2/1	3/1	1/2	2/2	3/2
35.	The basic approach to therapy and management of chronic rheumatic TMJ arthritis is composed of a systemic therapy of the underlying disorder complemented by therapy focusing specifically on the temporomandibular joint.	JIA/RA	V	Statement	**Strong consensus (100%)** **"yes" 6/6**			**Strong consensus (100%)** **"yes" 13/13**		
36.	In the cases of a chronic rheumatic TMJ arthritis an initial attempt at conservative therapy **shall** be made.	JIA/RA	IV	Expert consensus	**Strong consensus (100%)** **"shall" 6/6**			**Strong consensus (100%)** **"shall" 12/12, abstentions: 1**		
37.	The use of NSAIDs is to be approved for treatment of arthritic pain in the temporomandibular joint.	JIA/RA	IV	Statement	**Strong consensus (100%)** **"yes" 6/6**			**Strong consensus (100%)** **"yes" 13/13**		
38.	The application of topical NSAIDs according to Sidebottom's approach (four times daily, for four weeks) is to be approved.	JIA/RA	V	Statement	**Strong consensus (100%)** **"yes" 6/6**			**Strong consensus (100%)** **"yes" 11/11, abstentions: 2**		
39.	Together with the above recommendations, physiotherapy, including self exercises to be done by the patient (active movement training) are indicated:<br>- when jaw opening is restricted<br>- after exacerbation<br>- after arthroscopy<br>- after open joint surgery	JIA/RA	IV	Statement	**Strong consensus (100%)** **"yes" 6/6**			**Strong consensus (100%)** **"yes" 11/11, abstentions: 2**		
40.	Occlusal splints **should** be applied for relief of symptoms caused by TMJ arthritis and for improvement of clinical indicators.	JIA/RA	IIb	B	**Strong consensus (100%)** **"should" 6/6**			**Strong consensus (100%)** **"should" 12/12, abstentions: 1**		
41.	The application of distraction splints **may** reduce asymmetry and promote normalization of mandibular growth in JIA patients with unilateral TMJ arthritis.	JIA	IIIb	0	**Strong consensus (100%)** **"may" 6/6**			**Strong consensus (100%)** **"may" 12/12, abstentions: 1**		
42.	In JIA patients during growth, orthodontic functional devices (or activators) **may** be used for correction of dentofacial deformities (asymmetry, mandibular retrognathia, micrognathia, skeletal class II, skeletal open bite).	JIA	IIb	0				**Strong consensus (100%)** **"may" 11/11, abstentions: 2**		
43.	If malocclusion is the major leading symptom, or if surgical therapy is refused or contraindicated, and if TMJ arthritis is inactive or well-controlled by medication, orthodontic therapy **may** offer an alternative to surgery in adulthood, with the aim to correct dentoalveolar and/ or skeletal anomalies, as far as this is possible without surgical intervention.	RA	V	0	**Strong consensus (100%)** **"may" 6/6**			**Strong consensus (100%)** **"may" 12/12, abstentions: 1**		
44.	When ventilation therapy is required by means of "continuous positive airway pressure" (CPAP) therapy in the context of an obstructive sleep apnea syndrome (OSAS) as a result of retro- or micrognathia with chronic rheumatic TMJ arthritis, recourse to the nasal variety of CPAP therapy **may** be acceptable for relief of the temporomandibular joint, if also acceptable from a perspective of sleep medicine.	RA	IV	0				**Strong consensus (100%)** **"may" 12/12[13], abstentions: 1**		
45.	In the context of obstructive sleep apnea syndrome, as a result of retro- or micrognathia, and chronic rheumatic TMJ arthritis, a mandibular protrusion splint **may** be used for dilation and stabilization of the upper airways according to the standards of the German S3 Guideline Nr. 063/001	RA	IV	0				**Strong consensus (100%)** **"may" 12/12[14] abstentions: 1**		

[13] Two voting members out of the 12/12 who voted in favour of "may" also voted in favour of "should", one voting member out of the 12/12 who voted in favour of "may" also voted in favour of "shall"
[14] Two voting members out of the 12/12 who voted in favour of "may" also voted in favour of "should"

	Recommendation/Statement	Applicable for	LoE	GoR	1/1	2/1	3/1	1/2	2/2	3/2
	"Non-Restful Sleep/ Sleep Disorders - Sleep-Related Respiratory Disorders".									
46.	Easing the strain on the temporomandibular joint, e.g. by adopting a soft diet, in patients with chronic rheumatic TMJ arthritis of all age groups, and optimization of existing removable dentures for improvement of occlusion in chronic rheumatic TMJ arthritis in adults, are additional therapy approaches for symptom reduction and relief.	JIA/RA	IV	Statement	**Strong consensus (100%)**<br><br>**"yes" 6/6**			**Strong consensus (100%)**<br><br>**"yes" 12/12, abstentions: 1**		
47.	Insufficient response to, or failure of, medication therapy is considered as an indication for the application of IACIs in the context of an affection of the TMJ in rheumatoid arthritis (RA).	RA	V	Statement	**Strong consensus (100%)**<br><br>**"yes" 6/6**			**Strong consensus (100%)**<br><br>**"yes" 12/12, abstentions: 1**		
48.	Due to the risk of severe complications (chondrotoxicity,), the guideline group does not recommend IACI in cases where the temporomandibular joint exclusively is affected, or if intended as an additional measure during medication therapy, or for bridging during transition between medications in the context of a TMJ affection in RA.[15]	RA	V	Statement	**No consensus**<br><br>**"yes" 2/6, "no" 2/6, abstentions: 2/6**			**Consensus (88%)**<br><br>**"yes": 7/8, "no" 1/8, abstentions: 5**<br><br>Change in wording (Departure from vote on individual indication towards a "dissenting" overall statement)	**Strong consensus (100%)**<br><br>**"yes" 8/8, abstentions: 5**	
49.	Due to the risk of severe complications (chondrotoxicity), in the context of an TMJ affection of in RA IACI is to be applied as a one-time injection, if at all.	RA	IV	Statement		**No consensus**<br><br>**"shall" 1/6, "should" 2/6, "may" 3/6**	**Strong consensus (100%)**<br><br>**"yes" 6/6**	**Strong consensus (100%)**<br><br>**"yes" 12/12, abstentions: 1**		
50.	Application of IACI as a repeated or continuous therapy is absolutely to be avoided in the context of an affection of the TMJ in RA.	RA	IV	Statement		**Strong consensus (100%)**<br><br>**"yes" 6/6**		**Strong consensus (100%)**<br><br>**"yes" 12/12, abstentions: 1**		
51.	The concept of a one-time steroid injection according to Sidebottom and Salha's recommendation has been approved in the context of a TMJ affection in RA.	RA	V	Statement	**Strong consensus (100%)**<br><br>**"yes" 6/6**			**Strong consensus (100%)**<br><br>**"yes" 12/12, abstentions: 1**		
52.	Indications for the application of IACIs in the context of a TMJ affection in JIA currently are [16]:<br>- the temporomandibular joint exclusively is affected[17] (one-time injection)<br>- failure of medication therapy	JIA	V	Statement	**Strong consensus (100%)**<br><br>**"yes" 6/6**			**Strong consensus (100%)**<br><br>**"yes" 13/13**		
53.	Due to the risk of severe complications (disturbance of mandibular growth, heterotopic ossification) no indication for IACIs in JIA applies if intended as an additional measure	JIA	IV	Statement	**No consensus**			**Consensus (90%)**	**Strong consensus (100%)**	

[15] Adaptation of the wording in the Guideline to: Due to the possible risk of severe complications (chondrotoxic effect;) **no recommendation** could be made by the guideline group in favour of use of IACIs in adult patients if the TMJ exclusively is affected, for the purpose of supporting medicinal therapy or for bridging when transitioning between medications.

[16] According to therapy algorithms published until 12/2020

[17] In JIA patients, and if the TMJ only is affected, the use of IACIs has been and still is component in therapy algorithms. (Stoll et al. 2018, V/k++). Apart from a short-term therapeutic effect, these injections can also assist in making a distinction from symptoms whose origin is other than intra-articular. In view of potentially severe complications, however, there are indications of a paradigm shift (limitation to cases refractory to therapy) (Resnick et al. 2018, V/k+). In this context, the statement should always be assessed in the light of current developments.

	Recommendation/Statement	Applicable for	LoE	GoR	1/1	2/1	3/1	1/2	2/2	3/2
	during medication therapy or for bridging during transition between medications[18].				**"yes" 2/6, "no" 2/6, abstentions: 2/6**			**"yes" 9/10, "no" 1/10, abstentions: 3**<br><br>Change in wording (Departure from vote on individual indication towards a "dissenting" overall statement)	**"yes" 10/10, abstentions: 3**	
54.	Due to the risk of severe complications (disturbance of mandibular growth, heterotopic ossification) IACI in the context of TMJ affection in JIA is to be applied as a one-time injection – if at all.	JIA	IIIb	Statement		**No consensus**<br><br>**"shall" 1/6, "should" 2/6, "may" 3/6**<br><br>Change in wording ("Recommendation" replaced by "Statement")	**Strong consensus (100%)**<br><br>**"yes" 6/6**	**Strong consensus (100%)**<br><br>**"yes" 13/13**		
55.	Due to the risk of severe complications (disturbance of mandibular growth, heterotopic ossification) IACI in the context of JIA **"should"** be restricted to cases which are refractory to therapy.	JIA	V	B		**Strong consensus (100%)**<br><br>**"should" 6/6**		**Strong consensus (100%)**<br><br>**"should" 13/13**		
56.	Application as a regularly repeated or continuous therapy must absolutely be avoided in the context of a TMJ affection in JIA.	JIA	IIIb	Statement		**Strong consensus (100%)**<br><br>**"yes" 6/6**		**Strong consensus (100%)**<br><br>**"yes" 13/13**		
57.	Intraarticular infiltration of a local anesthetic, for temporary pain reduction and for the purpose of identification of the origin of the pain, according to the recommendations of Sidebottom and colleagues **may** be applied.	RA	V	0	**Strong consensus (100%)**<br><br>**"yes" 6/6[19]**			**Strong consensus (100%)**<br><br>**"may" 11/11[20], abstentions: 2**		
58.	If response to physiotherapy is incomplete, infiltration of the masseter and/ or temporalis muscle with long-acting local anesthetics or botulinum toxin for temporary reduction of myofascial pains and spasms according to the recommendations of Sidebottom and colleagues **may** be applied.	RA	V	0	**Strong consensus (100%)**<br><br>**"yes" 6/6[21]**			**Strong consensus (100%)**<br><br>**"may" 11/11[22], abstentions: 2**		
59.	In order to be able to counteract and avoid the risk of severe long-term sequelae, such as condylar resorption and ankylosis of the temporomandibular joint without delay, doctors and dentists involved in the therapy **shall** consult with an OMFS early in case of insufficient response to medicinal and conservative dental therapy.	JIA/RA	V	Expert consensus	**Strong consensus (100%)**<br><br>**"shall" 6/6**			**Strong consensus (100%)**<br><br>**"shall" 13/13**		
60.	With low risk of complications, low costs and well-tolerated, minimally invasive procedures **should** be used if conservative therapy fails, for further diagnostics, temporary pain reduction and improvement of TMJ function in chronic rheumatic TMJ arthritis.	JIA/RA	IIb	B	**Consensus (83%)**<br><br>**5/6 "should", 1/6 "may"**<br><br>Change in wording (addition)	**Consensus (83%)**<br><br>**5/6 "should", 1/6 "may"**		**Strong consensus (100%)**<br><br>**"should" 13/13**		

---

[18] Adaptation of the wording in the Guideline to: Due to the possible risk of severe complications (disturbance of mandibular growth, heterotopic ossification;) **no recommendation** could be made by the guideline group in favour of use of IACIs in JIA patients for the purpose of supporting medicinal therapy or for bridging when transitioning between medications

[19] Agreed upon in the exclusive OMS rounds as a "statement", converted into a "recommendation" in the interdisciplinary rounds

[20] One voting member out of the 11/11 who voted in favour of "may" also voted in favour of "should"

[21] Agreed upon in the exclusive OMS rounds as a "statement", converted into a "recommendation" in the interdisciplinary rounds

[22] Two voting members out of the 11/11 who voted in favour of "may" also voted in favour of "should"

	Recommendation/Statement	Applicable for	LoE	GoR	1/1	2/1	3/1	1/2	2/2	3/2
61.	If conservative therapy fails, arthrocentesis with joint lavage **may** be performed for reduction of pain and improvement of mandibular mobility.	JIA/RA	Ib	0	**Strong consensus (100%)** "may" 6/6			**Strong consensus (100%)** "may" 12/12, abstentions: 1		
62.	Arthrocentesis with joint lavage **may** be performed in combination with the intraarticular injection of corticosteroid (one-time injection).	JIA/RA	IIb	0	**Strong consensus (100%)** "may" 6/6			**Strong consensus (100%)** "may" 12/12, abstentions: 1		
63.	If conservative therapy fails, arthroscopy with joint lavage and lysis of adhesions, if necessary, **should** be performed for temporary reduction of pain and improvement of mandibular mobility.	JIA/RA	IIb	B	**Strong consensus (100%)** "should" 6/6			**Strong consensus (100%)** "should" 11/11, abstentions: 2		
64.	An approach of, if necessary, repeat application of arthroscopy with joint lavage for improvement of patient well-being comfort has been approved.	JIA/RA	V	Statement	**Strong consensus (100%)** "yes" 6/6			**Strong consensus (100%)** "yes" 11/11, abstentions: 2		
65.	A possible combination of arthroscopy and one-time intraarticular injection of corticosteroids has been approved.	JIA/RA	IV	Statement	**Strong consensus (100%)** "yes" 6/6			**Strong consensus (100%)** "yes" 11/11, abstentions: 2		
66.	In case of failure of all conservative and minimally invasive therapy options, and if the patient is expected to profit from the procedure symptomatically, functionally or aesthetically, open surgical procedures **shall** be considered.	JIA/RA	IV	Expert consensus	**Strong consensus (100%)** "shall" 6/6			**Consensus (91,6%)** "shall" 11/12, "may" 1/12, abstentions: 1		
67.	In cases of chronic rheumatic TMJ arthritis in adults and severe, long-term pain in combination with severe impairment of mandibular function and signs in medical imaging for synovitis or bony, arthritic damage in the meaning of early involvement, synovectomy **should** be performed.	RA	IIb	B	**Strong consensus (100%)** "should" 6/6[23]			**Consensus (91,6%)** "should" 11/12, "may" 1/12, abstentions: 1		
68.	In cases of severe damage to the disc, which, due to perforation or restricted mobility is beyond repair, discectomy **shall** be performed in case of clinical symptoms.	RA	V	Expert consensus	**Strong consensus (100%)** "shall" 6/6			**Consensus (90%)** "shall" 9/10, "may" 1/10, abstentions: 3		
69.	In adult patients with chronic rheumatic TMJ arthritis, an alloplastic total joint replacement **should** be preferable to an autologous one.	RA	IIIb	B	**Strong consensus (100%)** 6/6 "should"[24]			**Strong consensus (100%)** "should" 11/11, abstentions: 2		
70.	Due to increasing evidence of severe complications, especially unpredictable transplant growth, use of CCGs in adolescents at present is increasingly subject to controversy. The Grade 0-recommendation assigned is to be rated in context of the currently insufficient available data on therapeutic alternatives (notably total alloplastic joint replacement) to the CCG in children and adolescents.	JIA	IV	Statement		**Strong consensus (100%)** "yes" 6/6		**Strong consensus (100%)** "yes" 12/12, abstentions: 1		
71.	In cases of loss of function of the temporomandibular joint in adolescents with JIA, otherwise refractory to therapy (e.g. ankylosis), despite sometimes grave side effects, autologous reconstruction of the temporomandibular joint **may** be performed by means of a costochondral graft.	JIA	IV	0	**Consensus (83%)** 5/6 "may", 1/6 dissenting vote	**Consensus (83%)** 5/6 "may", 1/6 dissenting vote		**Strong consensus (100%)** "may" 12/12, abstentions: 1		

[23] In the OMS round, 1/6 votes also in favour of "shall"
[24] In the OMS round (see footnote 22) vote, 1/6 votes also in favour of "shall"

	Recommendation/Statement	Applicable for	LoE	GoR	1/1	2/1	3/1	1/2	2/2	3/2
					Change in wording (one supplementary note)					
72.	Following an autologous joint replacement by means of costochondral graft, follow-up examinations for early detection of potential complications and for possible further therapy design **shall** be performed until completion of mandibular growth.	JIA	IV	Expert consensus	**Strong consensus (100%)**<br><br>**"shall" 6/6**			**Strong consensus (100%)**<br><br>**"shall" 13/13**		
73.	In cases of severe deformations which cannot be controlled by conservative methods, an underlying disease which is inactive or well-controlled, and sufficient remaining condylar mass, orthognathic surgery **should** be considered.	JIA	IV	B	**Strong consensus (100%)**<br><br>**"should" 6/6**			**Strong consensus (100%)**<br><br>**"should" 12/12, abstentions: 1**		
74.	As a general rule, orthognathic-surgical procedures and/or autologous replacements will always carry the risk of renewed recurrence of dysgnathia caused by arthritis. Multiple repeat surgeries due to such relapses, often to be observed in the past, are to be avoided given the fact that alternatives which provide long-term durability and stability have become available.	JIA	V	Comment		**Strong consensus (100%)**<br><br>**"yes" 6/6**		**Strong consensus (100%)**<br><br>**"yes" 12/12, abstentions: 1**		
75.	Under specific circumstances (e.g. grave psychic distress), orthognathic surgery in JIA patients is also an option during growth.	JIA	IV	Statement	**Strong consensus (100%)**<br><br>**"yes" 6/6**			**Strong consensus (100%)**<br><br>**"yes" 12/12, abstentions: 1**		
76.	In JIA patients with skeletal deformities and TMJ involvement, bilateral sagittal split osteotomy (BSSO)[25] is a procedure basically suitable for mandibular forward displacement.	JIA	IV	Statement	**Strong consensus (100%)**<br><br>**"yes" 6/6**			**Strong consensus (100%)**<br><br>**"yes" 11/11, abstentions: 2**		
77.	In JIA patients with skeletal deformities and TMJ involvement, horizontal mandibular distraction osteogenesis[26] is a procedure basically suitable for mandibular forward displacement.	JIA	IV	Statement	**Strong consensus (100%)**<br><br>**"yes" 6/6**			**Strong consensus (100%)**<br><br>**"yes" 11/11, abstentions: 2**		
78.	The approach of Nørholt and colleagues, i.e. application of NSAIDs one hour prior to distractor activation and use of an occlusal splint to shift the load from the temporomandibular joint to the teeth has been approved.	JIA	IV	Statement	**No consensus**<br><br>**"yes" 3/4, "no" 1/4, abstentions: 2**			**No consensus**<br><br>**"yes" 6/6, abstentions: 7**		
79.	For skeletal deformities in the context of TMJ involvement in JIA, or as a result of JIA, Le Fort I osteotomy[27] for correction of occlusion and repair of an anterior open bite after the end of the growth phase, is a possible option in selected cases, provided the underlying disease is inactive/well controlled or adequately managed, as otherwise there is a risk of recurrence. Furthermore, factors such as sufficient posterior airway space (PAS) and the basic dentofacial aspects of orthognathic surgery need to be considered.	JIA	IV	Statement	**Consensus (83%)**<br><br>**"yes" 5/6, "no" 1/6**<br><br>Change in wording (based on comments by one voting member)	**Strong consensus (100%)**<br><br>**"yes" 6/6**		**Strong consensus (100%)**<br><br>**"yes" 11/11, abstentions: 2**		
80.	For skeletal deformities in the context of TMJ involvement in JIA, or as a result of JIA, genioplasty[28] for occlusal correction and optimization of facial aesthetics after reaching skeletal maturity constitutes an adequate therapy option in principle.	JIA	IV	Statement	**Strong consensus (100%)**<br><br>**"yes" 6/6**			**Strong consensus (100%)**<br><br>**"yes" 11/11, abstentions: 2**		

[25] Provided the underlying disease is inactive/well controlled or adequately managed, as otherwise there is a risk of recurrence.
[26] Provided the underlying disease is inactive/well controlled or adequately managed, as otherwise there is a risk of recurrence.
[27] Provided the underlying disease is inactive/well controlled or adequately managed, as otherwise there is a risk of recurrence.
[28] Provided the underlying disease is inactive/well controlled or adequately managed, as otherwise there is a risk of recurrence.

	Recommendation/Statement	Applicable for	LoE	GoR	1/1	2/1	3/1	1/2	2/2	3/2
81.	Figure 3: Clinical algorithm for correction of skeletal deformities in JIA, with modifications according to (Resnick et al. 2019, IV/k++)	JIA	IV	Diagram		**Strong consensus (100%)**<br><br>**"yes" 6/6**		**Strong consensus (100%)**<br><br>**"yes" 11/11, abstentions: 2**		
